# Gastric cancer peritoneal metastasis related signature predicts prognosis and sensitivity to immunotherapy in gastric cancer

**DOI:** 10.1111/jcmm.17922

**Published:** 2023-08-21

**Authors:** YuQin Sun, YueQing Chen, Wei Zhuang, ShunYong Fang, QiuXian Chen, MingQiao Lian, ChenBin Lv, JianMing Weng, Ran Wei, Yao Lin, LiSheng Cai, QingShui Wang

**Affiliations:** ^1^ Department of Gastrointestinal Surgery Zhangzhou Affiliated Hospital of Fujian Medical University Zhangzhou China; ^2^ Department of Pathology Zhangzhou Affiliated Hospital of Fujian Medical University Zhangzhou China; ^3^ Department of Urology The Second Affiliated Hospital of Fujian Medical University Quanzhou China; ^4^ Changzhou Key Laboratory of Respiratory Medical Engineering, Institute of Biomedical Engineering and Health Sciences, School of Medical and Health Engineering Changzhou University Changzhou China; ^5^ Central Laboratory at the Second Affiliated Hospital of Fujian University of Traditional Chinese Medical UniversityMedicine, Fujian‐Macao Science and Technology Cooperation Base of Traditional Chinese Medicine‐Oriented Chronic Disease Prevention and Treatment, Innovation and Transformation Center, Fujian University of Traditional Chinese Medicine Fuzhou China; ^6^ College of Life Sciences, Fujian Normal University Fuzhou China

**Keywords:** fibroblasts, gastric cancer, immunotherapy, peritoneal metastases, tumour microenvironment

## Abstract

Gastric cancer peritoneal metastases (GCPM) is a leading cause of GC‐related death. Early detection of GCPM is critical for improving the prognosis of advanced GC. Differentially expressed genes (DEGs) were identified in the GSE62254 database to distinguish between GCPM and non‐GCPM. The gastric cancer peritoneal metastases signature (GCPMs) was developed using DEGs. We analysed the effectiveness of GCPMs as indicators for prognosis, chemotherapy, and immune therapy response in GC patients. Subsequently, we analysed the correlation between GCPMs and immune microenvironment as well as immune escape in GC patients. Random forest model and immunohistochemistry was utilized to identify the crucial genes that can aid in the diagnosis of GCPM. We identified five DEGs and utilized their expression to construct GCPMs. Patients with high GCPMs had a higher likelihood of a poor prognosis, while those with low GCPMs appeared to potentially benefit more from chemotherapy. GCPMs were a dependable marker for predicting the response to immunotherapy. Additionally, GCPMs was found to be significantly linked to stromal score and cancer‐associated fibroblasts. SYNPO2 has been identified as the gene with the highest significance in the diagnosis of GCPM. Immunohistochemistry suggests that SYNPO2‐positive expression in tumour cells, fibroblasts, inflammatory cell may be associated with promoting peritoneal metastasis in GC. GCPMs have shown to be a promising biomarker for predicting the prognosis and response of GC patients to chemotherapy and immunotherapy. The use of GCPMs for individual tumour evaluation may pave the way for personalized treatment for GC patients in the future.

## 1 INTRODUCTION

Gastric cancer is highly prevalent in eastern Asia, ranking as the fifth most commonly diagnosed cancer and the third leading cause of cancer‐related death.^1^ Because of the lack of personalized targets, treatment for GC is currently selected mainly based on TNM staging system; however, there is a survival paradox at the same stage frequently, which traditional clinicopathological features fail to explain. Gastric cancer is known for its significant genotypic heterogeneity.^2^ Through its analysis of gastric cancer, TCGA has classified the disease into four distinct subtypes, which are microsatellite instable (MSI), Epstein–Barr virus‐infected (EBV), chromosomally unstable (CIN) and genomically stable (GS).^3^ While The Asian Cancer Research Group (ACRG) has identified four subtypes of gastric cancer, which include MSI, Microsatellite stable/Epithelial‐to‐Mesenchymal Transition (MSS/EMT), MSS/TP53+, and MSS/TP53−.^4^ These subtypes have been linked to specific treatment decisions and show distinct clinical outcomes in patients with gastric cancer.

Gastric Cancer peritoneal metastasis (GCPM) is the most common types of GC recurrence associated with poor survival.^5^ Conventional imaging techniques may not accurately detect or measure GCPM, diagnostic laparoscopy with peritoneal lavage cytology can help identify disseminated cancer cells within the peritoneal cavity for a more accurate diagnosis.^6^ Gastric cancer patients who are at high risk of developing GCPM may not show significant benefits from conventional therapies.^7^ It is recommended to adopt a combination of systemic therapy and peritoneal‐directed treatment strategies, such as intraperitoneal chemotherapy, which have demonstrated an encouraging trend towards improved disease outcomes. GCPM has been linked to various clinicopathological characteristics, genetic mutations and molecular signatures.^8^, ^9^, ^10^ However, the usefulness of these histopathology and molecular classifications in clinical practice remains limited. The current selection of treatment for gastric cancer is predominantly based on disease stage, as there are limited individualized targets available. To improve the accuracy of risk stratification and enhance survival prediction after curative resection, there is an urgent need to develop more precise biomarkers related to peritoneal metastasis recurrence. These biomarkers can aid in individualized therapeutic interventions, even before surgery and help optimize treatment strategies.

In this study, we aimed to develop a Gastric Cancer Peritoneal Metastases Signature (GCPMS) that could predict peritoneal metastasis, survival and responses to immunotherapy and chemotherapy. Our findings demonstrated that GCPMS served as an independent prognostic factor for gastric cancer and was strongly associated with response to immunotherapy and chemotherapy. It's worth noting that GCPMS was also validated for its ability to identify patients at risk of peritoneal metastasis across multiple cohorts of gastric cancer.

## 2 METHODS

### 2.1 Clinical data acquisition and extraction

To obtain the necessary data for our study, we downloaded transcriptome RNA‐sequencing data along with clinical information pertaining to gastric cancer based on the TCGA database and obtained the gene expression profiles and matched clinical information for the GSE62254,^4^ GSE13861^11^ and GSE26253^12^ (SMC cohort) databases from the GEO database. Additionally, also collected information from the SMC cohort,^13^ comprising of 432 patients who had undergone curative gastrectomy and had received the INT‐0116 treatment regimen (which includes 5‐fluorouracil/leucovorin and radiation) as an adjuvant therapy. This dataset could provide valuable insights into the effectiveness of this treatment approach in gastric cancer patients who have undergone surgical resection. We obtained the PD‐L1 treatment cohort for gastric cancer (KIM cohort) from the Tumour Immune Dysfunction and Exclusion (TIDE) database, which is accessible at http://tide.dfci.harvard.edu.^14^ This dataset could provide valuable information on the effectiveness of PD‐L1 treatment in gastric cancer patients. We employed the identical methodology for retrieving microarray gene expression data as outlined in our previous research.^15^, ^16^

### 2.2 Identification of the GCPMs genes

In the study, GSE62254 database was used to identify GCPMs genes by comparing the DEGs between GCPM patients (*n* = 61) and non‐GCPM patients (*n* = 239). We conducted the analysis using the “limma” R package, and set the significance threshold at |log2FC|≥0.585 and False Discovery Rates (FDR) < 0.05. The average mean of the mRNA expression of DEGs genes was calculated as GCPMs.

### 2.3 Genetic alteration and survival prognosis analysis

We downloaded the somatic mutation data of gastric cancer from TCGA using the UCSC XENA website.^17^ To compare the overall survival (OS) and disease‐free survival (DFS) rates among patients, we utilized Kaplan–Meier curves along with the log rank test. The “survival” R package was used to determine the optimal cutoff points.

### 2.4 Immune infiltration analysis

To estimate the immune infiltration in gastric cancer, we employed the CIBERSORT algorithm. We analysed the correlation between GCPMs and immune cells using both Spearman and distance correlation analysis. Additionally, we assessed the stromal score of each sample using the “ESTIMATE” R software package.

### 2.5 Drug sensitivity analysis

To identify potential molecular compounds for targeted therapy, we conducted drug sensitivity analysis using GSCALite website to evaluate the drug sensitivity of GCPMs genes (http://bioinfo.life.hust.edu.cn/web/GSCALite/).^18^

### 2.6 Patients and specimens

A total of 30 gastric cancer tissues (15 GCPM patients and 15 non‐GCPM patients) from Zhangzhou Affiliated Hospital of Fujian Medical University between November 2019 and November 2021 were collected. The project was approved by the Research Ethics Committee of Zhangzhou Affiliated Hospital of Fujian Medical University. All patients involved in the study provided written informed consent after approval by the relevant institutional protocol.

### 2.7 Immunohistochemistry (IHC) staining analysis

To measure the protein expression of SYNPO2 in gastric cancer, we conducted IHC staining analysis using a standard immunoperoxidase staining procedure. Specifically, slides were incubated with an anti‐SYNPO2 antibody (bs‐8743R, Bioss, diluted 1:400). To ensure the accuracy of the IHC staining scores for SYNPO2, we had two independent pathologists evaluate them. SYNPO2 expression was evaluated on tumour, fibroblasts and inflammatory cells according to the CPS (combined positive score). CPS is the sum of SYNPO2–stained cells' number (tumour cells, fibroblasts, inflammatory cell) divided by the total number of viable tumour cells, multiplied by 100, as in the formula below: CPS = Number of SYNPO2 stained cells (tumour cells, fibroblasts, inflammatory cell) × 100/Total number of viable tumour cells. This CPS score was then calculated for each case.

### 2.8 Single‐cell level analysis

Tumour Immune Single‐cell Hub 2 (http://tisch.comp‐genomics.org/home/),^19^ also known as TISCH2, is a scRNA‐seq database that focuses on the tumour microenvironment (TME). This database provides comprehensive cell‐type annotation at the single‐cell level, allowing for the exploration of TME across various cancer types.

### 2.9 LASSO and random forest analysis

To obtain diagnostic markers for GCPMs, we utilized LASSO logistic regression for the purpose of feature selection. Random forest models employed the “randomforest” package.

### 2.10 Statistical analysis

Statistics analysis was performed with the student's *t*‐test. The Kaplan–Meier method was used to calculate OS and DFS, while the log‐rank test was utilized to examine differences between groups. We considered a *p*‐value threshold of <0.05 to determine statistical significance.

## 3 RESULTS

### 3.1 The landscape of GCPMs in gastric cancer

**FIGURE 1:**
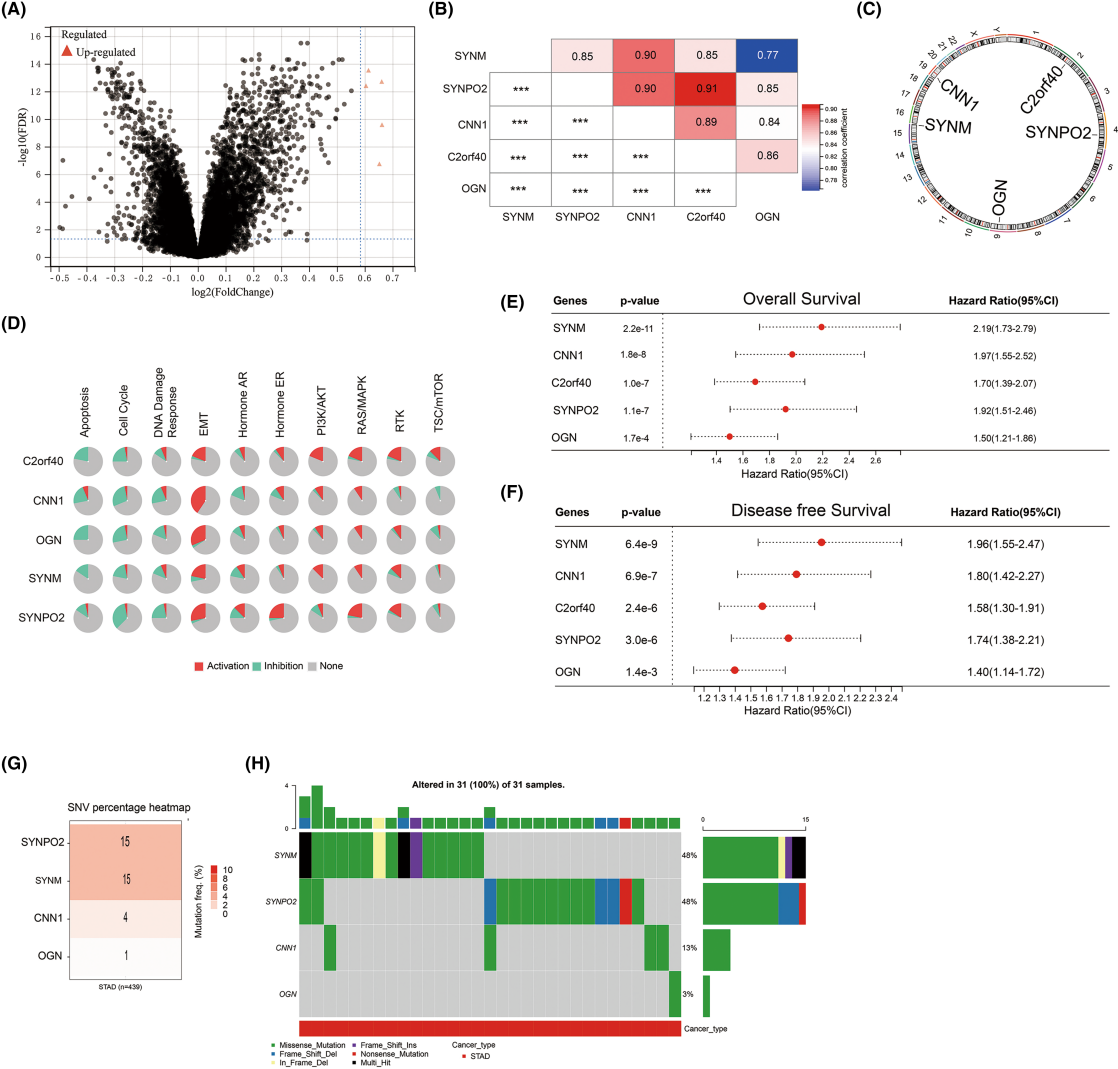
Landscape of genetic variation and correlation of GCPMs in gastric cancer. (A) volcano plot was generated to visualize the differentially expressed genes (DEGs) between GCPM tumours and non‐GCPM tumours utilizing data from the GSE62254 database. (B) Spearman's correlation analysing the relation between five genes using GSE62254 database. (C) The chromosomal locations of the five genes were determined and their distribution was analysed. (D) The associations between the five genes and relevant biological pathways. (E) OS analysis of the five genes in gastric cancer based on GSE62254 database. (F) DFS analysis of the five genes in gastric cancer based on GSE62254 database. (G) The number of mutations present in four specific genes in cases of gastric cancer using TCGA database. (H) Oncoplots showing the mutation landscape of four genes in gastric cancer patients from TCGA database. Each column represents an individual sample.

To identify the gene expression signature associated with GCPM, we compared the gene expression profiles of GCPM (*n* = 61) and non‐GCPM (*n* = 239) based on GSE62254 database (Figure 1A). Compared with non‐GCPM group, five genes (SYNM, SYNPO2, CNN1, C2orf40 and OGN) were up‐regulated in GCPM group. A Spearman correlation analysis revealed significant correlations between these five genes and others (Figure 1B). The average mean of the mRNA expression of DEGs genes was calculated as GCPMs. Additionally, we analysed the chromosomal distribution of these five genes and found that they were exclusively located in the autosomes (Figure 1C). The functional investigation of these five genes demonstrated their ability to activate the EMT, PI3K/AKT pathway, and RAS/MAPK pathway, while also inhibiting the apoptosis and cell cycle (Figure 1D). The analysis of survival rates indicated that high expression of all five genes were significantly associated with poor OS and DFS (Figure 1E,F). Given the crucial impact of gene mutation and copy number variation on the progression of cancer, our analysis included a CNV investigation using the TCGA database. Our results revealed that SYNPO2 and SYNM displayed the highest frequency of CNV among the five genes, followed by CNN1 and OGN. On the other hand, the C2orf40 gene did not exhibit any mutations (Figure 1G,H).

### 3.2 Characterization of molecular features of GCPMs‐Low and GCPMs‐High subtypes

**FIGURE 2:**
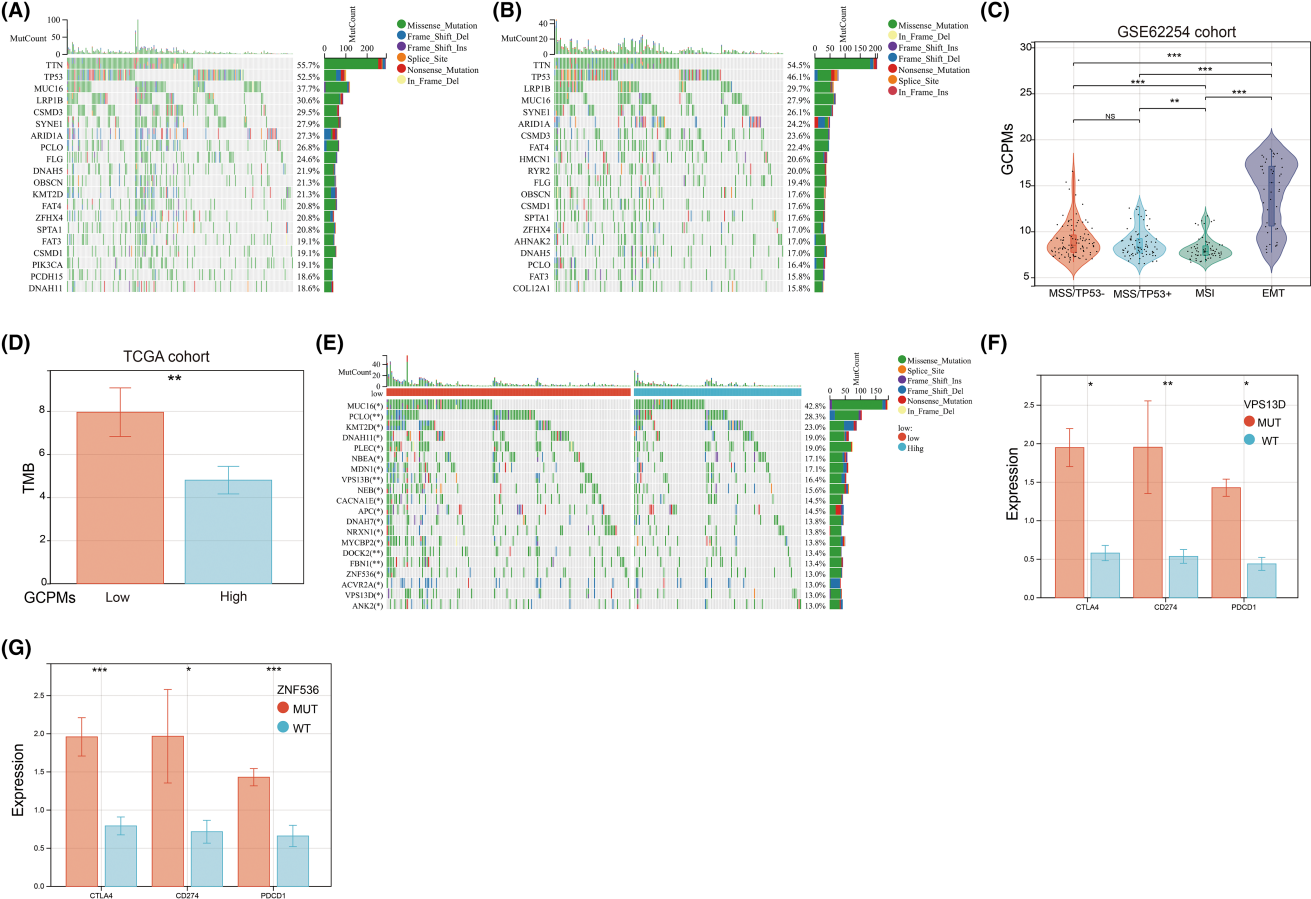
Relationship among the GCPMs, genomic alterations, and molecular subtypes in gastric cancer. (A,B) Oncoplots showing landscapes of genomic alterations in both (A) low and (B) high GCPMs subtypes, respectively. (C) The correlations between the GCPMs and molecular subtypes of gastric cancer in GSE62254 database. (D) Differences in tumour mutation burden between low GCPMs and high GCPMs subtypes. (E) Top 15 genes with the highest mutation frequency related to the GCPMs based on TCGA database. (F,G) VPS13D and ZNF536 mutations distinctly facilitated expression of immune checkpoints (CTLA4, CD274 and PDCD1). *p < 0.05; **p < 0.01; ***p < 0.001.

We further conducted a genomic alteration analysis on the GCPMs‐Low and GCPMs‐High subtypes and noted that the top 20 genes with the highest mutation frequency exhibited a general similarity between the GCPMs‐Low and GCPMs‐High subtypes. However, the GCPMs‐Low subtype had a relatively higher mutation frequency for each gene compared to the GCPMs‐High subtype (Figure 2A,B). Our analysis of GSE62254 revealed that the EMT subtype had the highest GCPMs, while the MSI subtype had the lowest GCPMs (Figure 2C). Due to the production of immunogenic neoantigens, tumour mutation burden (TMB) has become a promising biomarker for immunotherapy. To investigate further, we analysed the difference in TMB between GCPMs‐Low and GCPMs‐High subtypes. Interestingly, our findings revealed that the GCPMs‐Low subtype had a significantly higher level of TMB compared to the GCPMs‐High subtype (Figure 2D). Then we identified the top 15 mutated genes that were positively associated with GCPMs in the TCGA database. (Figure 2E). To explore the potential for immunotherapy response, we analysed the relationship between the expression levels of immune checkpoints and the mutated genes. Our findings demonstrated that patients with mutations in VPS13D or ZNF536 presented significantly higher levels of immune checkpoints (CTLA4, CD274 and PDCD1) compared to those with wild type VPS13D or ZNF536 (Figures 2F,G).

### 3.3 The correlation between GCPMs and clinical features and prognosis

**FIGURE 3:**
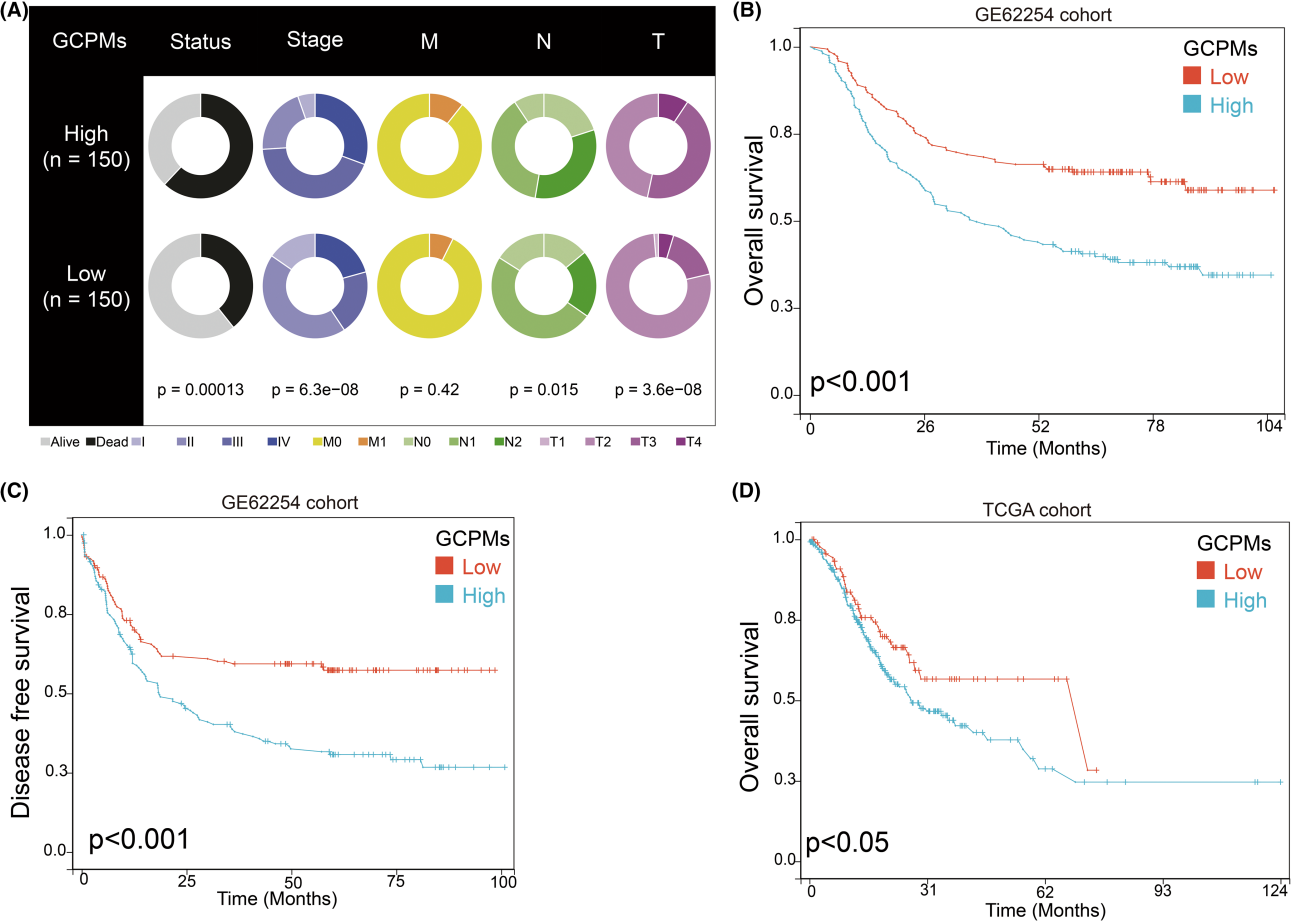
Correlation between GCPMs subtypes and clinicopathological features and prognosis in GSE62254 and TCGA databases. (A) Different clinicopathological features of low GCPMs and high GCPMs subtypes in GSE62254 database. (B–D) Survival analysis showing the differences of OS and DFS between low GCPMs and high GCPMs.

We then conducted a comparison of clinical characteristics between GCPMs‐low and GCPMs‐high subtypes using the GSE62254 database. Our analysis revealed that the proportion of patients in Stage III and IV was significantly higher in the GCPMs‐high subtype (Figure 3A). Our analysis of clinical outcomes revealed that patients with high GCPMs had a significantly poorer prognosis compared to those with low GCPMs, as evidenced by both OS and DFS rates (Figure 3B–D). These results strongly suggest that GCPMs based on our findings, it is highly probable that GCPMs could be used as a reliable prognostic indicator for patients with gastric cancer.

### 3.4 GCPMs predicted the response to adjuvant therapy in gastric cancer

**FIGURE 4:**
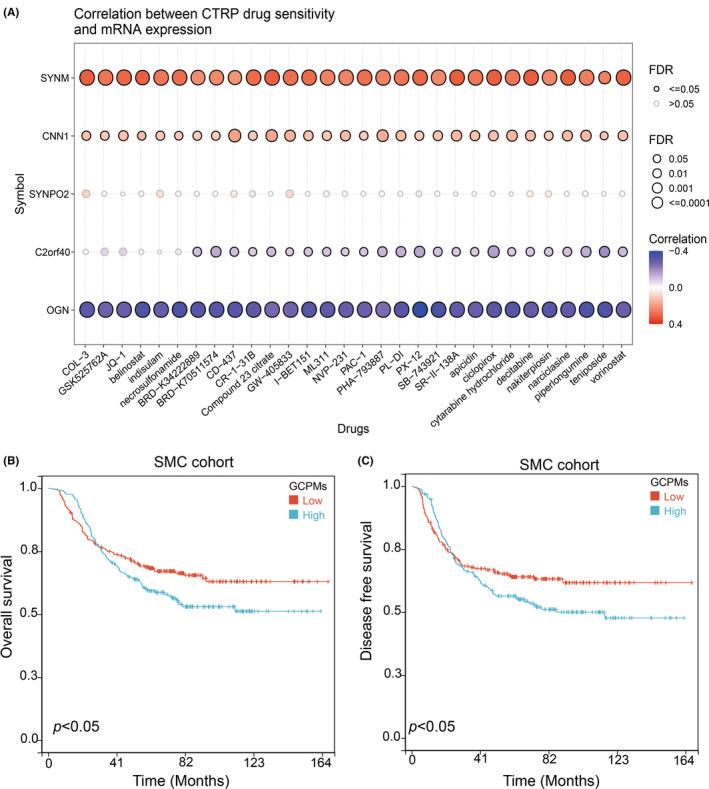
Prediction and correlation of the sensitivity to chemotherapy drugs in gastric cancer. (A) The correlation between GDSC drug sensitivity and five genes expression. (B,C) Kaplan–Meier survival plots showing the differences of (B) overall survival and (C) disease free survival between low GCPMs and high GCPMs.

Previous clinical research has consistently demonstrated that the addition of adjuvant chemotherapy can significantly enhance the prognosis of patients with advanced gastric cancer, in comparison to surgical treatment alone. Despite the benefits of adjuvant chemotherapy, drug resistance remains a significant obstacle to achieving positive treatment outcomes. To explore potential mechanisms of drug resistance, we evaluated the relationship between drug sensitivity and the expression of five GCPMs genes using the Cancer Therapeutics Response Portal (CTRP) database. According to our results, higher levels of SYNM and CNNN1 were found to be positively associated with increased IC50 values of cancer therapy drugs, whereas the expression of OGN and C2orf40 exhibited an opposite correlation (Figure 4A). These results suggest that these findings could have important clinical implications in guiding the formulation of chemotherapy regimens. Furthermore, we also examined the relationship between GCPMs and the response to adjuvant chemoradiotherapy in a group of 432 patients who underwent uniform chemoradiotherapy (combination of 5‐fluorouracil/leucovorin with radiation) following surgical intervention (SMC cohort). The findings indicated that patients having low GCPMs experienced greater benefits in terms of OS and DFS, as compared to those having high GCPMs (Figure 4B,C). In conclusion, GCPMs emerged as a reliable tool for predicting the response of gastric cancer patients to adjuvant therapy. Notably, individuals classified under the low GCPMs subtype may gain significant benefits from receiving adjuvant chemotherapy.

### 3.5 GCPMs predicted the response to immunotherapy in gastric cancer

**FIGURE 5:**
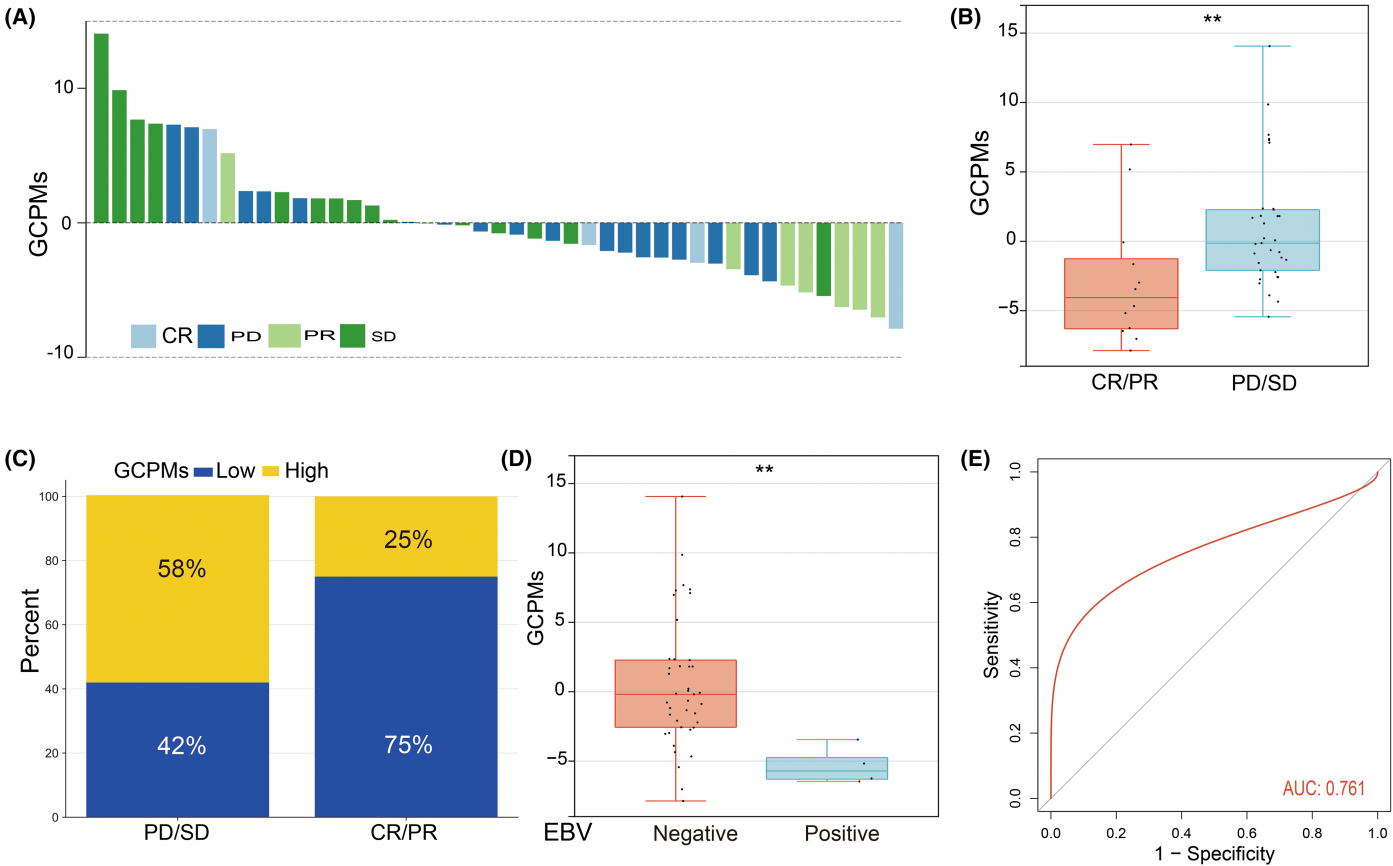
Prediction of the response to immune checkpoint blockade treatment. (A) The correlation of GCPMs with response to immunotherapy in KIM cohort. CR (complete response), PD (progressive disease), PR (partial response), SD (stable disease). (B) Difference in GCPMs between PD/SD group and PR/CR group. (C) The proportion of patients with different response to immunotherapy in two GCPMs subtypes. (D) Difference in GCPMs between EBV positive and negative status. (E) The predictive value of GCPMs in gastric cancer patients treated with immunotherapy. **, *p* < 0.01.

The emergence of immunotherapy, particularly the PD1/PDL1 checkpoint inhibitors, represents a significant milestone in cancer treatment. The monoclonal PD‐1 blockade has been granted approval for use as a primary treatment option for individuals with advanced or metastatic gastric cancer. Given the promising results of immunotherapy in gastric cancer, we conducted further evaluations of the predictive capabilities of GCPMs within the KIM cohort. Figure 5A displays the GCPMs of gastric cancer patients with varying therapeutic responses. Our findings demonstrated that individuals who had progressive or stable disease (PD/SD) had significantly higher GCPMs than those who had partial or complete response (PR/CR) (Figure 5B). It is worth noting that PR/CR group exhibited a prevalence of the GCPMs‐low subtype, accounting for 75% of cases, while the PD/SD group had a higher occurrence (58%) of the GCPMs‐high subtype. As illustrated in Figure 5C, our results suggest that GCPMs could act as a negative predictor for the efficacy of immunotherapy in gastric cancer. In addition, it has been reported that EBV status is a crucial indicator for the effectiveness of immunotherapy. We observed that patients with negative EBV status had higher GCPMs compared to those with positive EBV status, as shown in Figures 5D. We calculated the area under the curve (AUC) of GCPMs and found it to be 0.761, as shown in Figure 5E. These results suggest that GCPMs may have potential predictive value for immunotherapy response in patients with gastric cancer. In summary, our study suggests that patients harbouring the GCPMs‐low subtype may have a higher likelihood of positive response to immunotherapy, while those with the GCPMs‐high subtype may have a lower chance of responding to this treatment. GCPMs may hold great potential as a reliable prognostic marker for predicting immunotherapy response in patients with gastric cancer.

### 3.6 GCPMs and tumour microenvironment in gastric cancer

**FIGURE 6:**
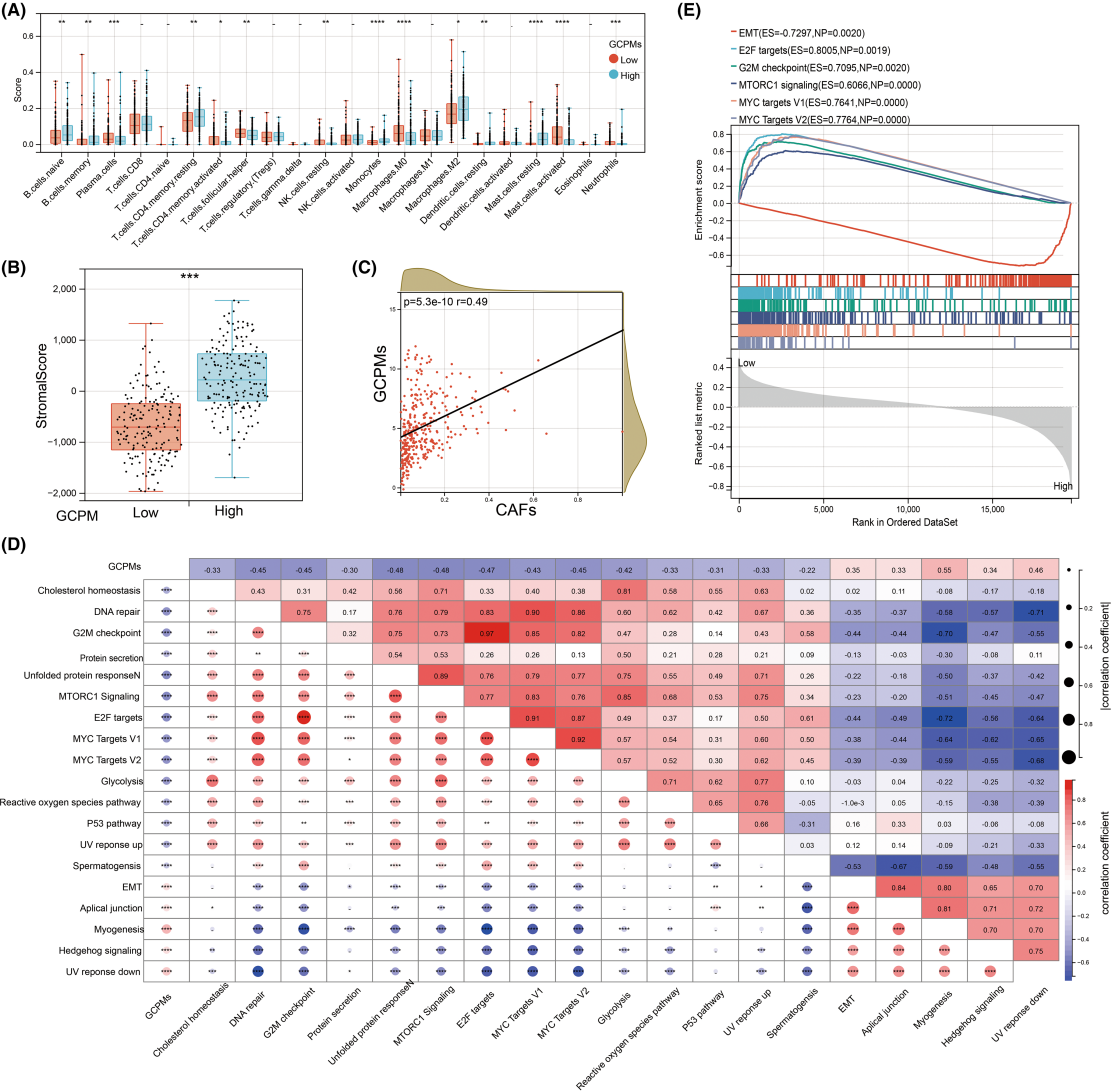
Correlation between the GCPMs and tumour microenvironment in gastric cancer. (A) Box plots illustrating the relationships between GCPMs subtypes and the infiltration of immune cells. (B) stromal score was significantly associated with GCPMs subtypes. (C) Spearman analysis of correlation between GCPMs and CAFs. (D) A correlate demonstrating correlations among GCPMs and the known gene signatures by Spearman analysis. (E) GSEA analysis of low and high GCPMs subtypes. *p < 0.05; **p < 0.01; ***p < 0.001; ****p < 0.0001.

TME plays a crucial role in both the progression of tumours and the efficacy of therapeutic interventions. To explore the correlation between GCPMs and the immune microenvironment, we examined the distribution of immune cells infiltrating both GCPMs‐low and GCPMs‐high subtypes. Our analysis revealed that the GCPMs‐high subtype showed markedly elevated levels of infiltration by B cells naive, B cells memory, T cells CD4 memory resting, Monocytes, Macrophages M2, Dendritic cells resting, Mast cells resting and Mast cells activated, GCPMs‐low subtype exhibited significantly higher infiltration of Plasma cells, T cells follicular helper, T cells CD4 memory activated, NK cells resting, Macrophages M0, Mast cells activated and Neutrophils (Figure 6A). Regarding non‐immune cells, our analysis revealed a significant correlation between GCPMs and cancer‐associated fibroblasts (CAFs) (Figure 6B). Given that CAFs have been shown to impede T cell infiltration and function within the tumour microenvironment, we investigated the association between GCPMs and stromal score, which reflects the abundance of stromal cells. Our analysis demonstrated a positive correlation between GCPMs and stromal score in gastric cancer (Figure 6C). To gain further insight into the potential role of GCPMs, we examined the correlation between GCPMs and established molecular signatures (Figure 6D). Our analysis revealed that several pathways were positively associated with GCPMs, including EMT, apical junction, myogenesis, Hedgehog and UV response down pathways (Figure 6D). In contrast, DNA repair, G2M checkpoint, MYC targets V1 and MYC targets V2 pathways were found to be negatively correlated with GCPMs. Furthermore, our analysis indicated that the EMT pathway was highly enriched in the GCPMs‐high subtype (Figure 6E). These findings support the notion that GCPMs may be closely associated with the tumour microenvironment of gastric cancer.

In recent years, single‐cell RNA sequencing (scRNA‐seq) has become a valuable tool in analysing the molecular characteristics of individual cells. One of the major benefits of scRNA‐seq is the ability to analyse gene expression profiles of individual cells, providing a more precise understanding of the tumour microenvironment compared to traditional bulk analysis methods. To investigate the function of GCPMs in the TME, we conducted an analysis of the GSE134520 and GSE167297 databases which were generated from scRNA‐seq studies of single cells collected from patients diagnosed with diffuse‐type gastric cancer. The purpose of this analysis was to enhance our comprehension of the role and manifestation tendencies of GCPMs within the tumour microenvironment. After quality control screening, the UMAP algorithm was used to cluster a total of 22,464 cells in the GSE167297 database into distinct nine cell clusters including CD8 T cells, B cells, plasma cells, dendritic cells, monocytes or macrophages, mast cells, endothelial cells, fibroblasts and epithelial cells, a total of 41,5544 cells in the GSE134520 database was divided into nine cell clusters including CD8 T cells, plasma cells, dendritic cells, mast cells, fibroblasts, myofibroblasts, epithelial cells, malignant cells, gland mucous and pit mucous. Then we examined the expression of five genes in the identified cell clusters using data from two databases, GSE134520 and GSE167297. Interestingly, we observed high expression levels of SYNM, SYNPO2, CNN1 and OGN genes in fibroblast cells, while C2orf40 gene expression was not detected (Figure S1). In summary, our comprehensive analyses have revealed the relationship between GCPMs and TME in gastric cancer. Based on these results, it can be inferred that GCPMs have a vital function in tumour immunity and metastasis and can also serve as a prognostic indicator for various types of adjuvant therapy in this ailment. These outcomes offer valuable perspectives regarding the potential practical uses of GCPMs in clinical settings.

### 3.7 Construction and validation of GCPMs diagnostic model

Next, we employed lasso logistic analysis to construct a diagnostic model for distinguishing GCPM patients from non‐GCPM patients. This model was developed using the GSE62254 database and identified two key‐gene signatures as potential diagnostic markers. The formula of diagnostic model constructed was as follow: Risk score = (−0.826 × expression level of SYNM)−(0.901 × expression level of SYNPO2). This formula calculates a risk score for each patient based on the expression levels of the two key‐gene signatures. The risk score can be used to classify patients as either GCPM or non‐GCPM, with higher scores indicating a higher likelihood of having GCPM. To evaluate the diagnostic model's effectiveness and accuracy, we performed ROC analysis on both the GSE62254 database and a validation dataset (GSE13861). The AUC values obtained from this analysis demonstrated that the diagnostic model was highly effective in identifying GCPM patients, with an AUC of 0.759 for GSE62254 and 0.701 for GSE13861 (Figure S2). These results highlight the diagnostic model's ability to accurately distinguish between GCPM and non‐GCPM patients, indicating its potential as a useful diagnostic tool in clinical settings.

### 3.8 SYNPO2 is an important gene for predicting GCPM

**FIGURE 7:**
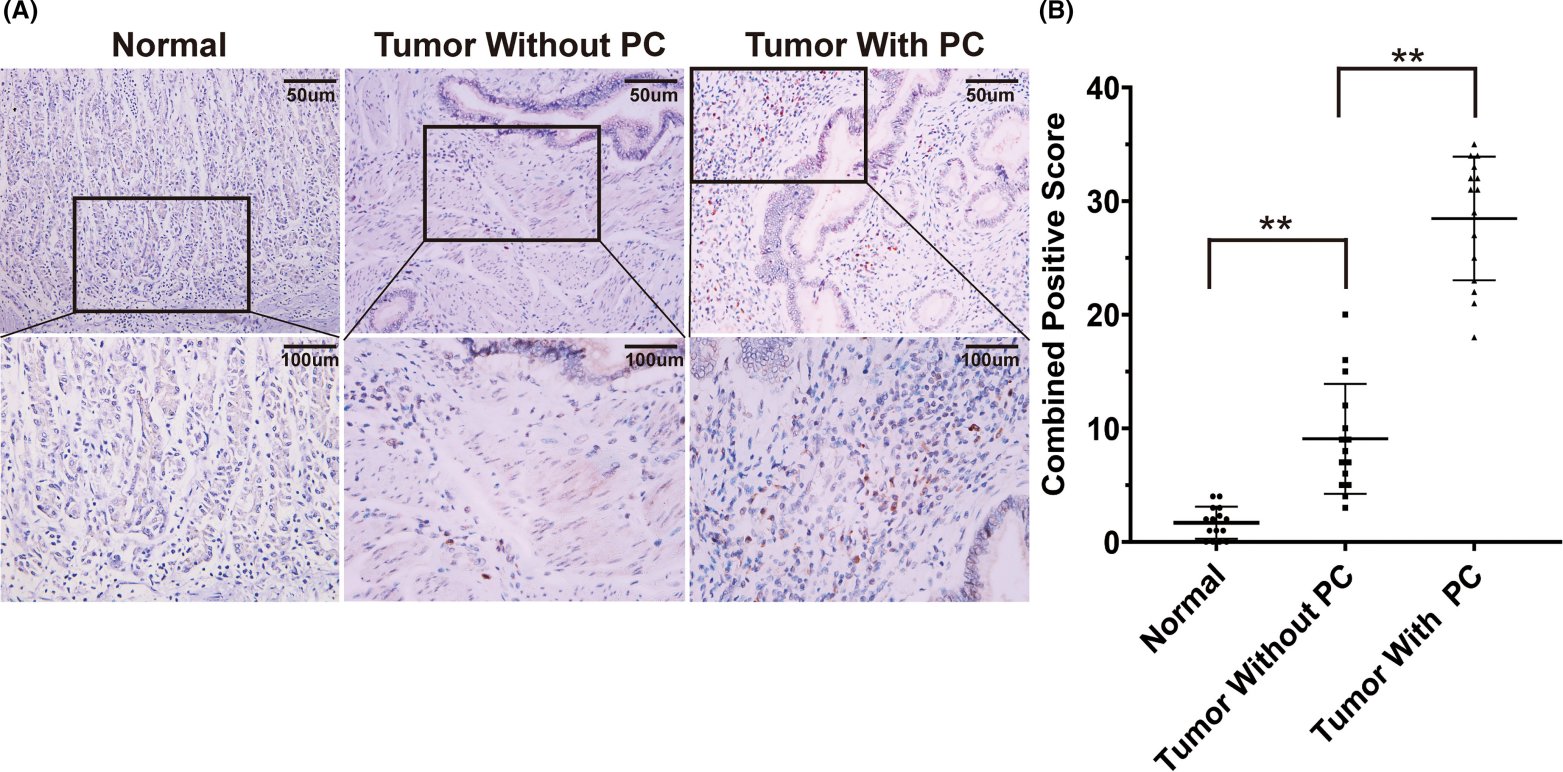
Immunohistochemical detection revealed the expression of SYNPO2 in GC.

After screening potential GCPM biomarkers, the random forest model was used to identify the most significant gene for diagnosing GCPM patients. Analysis using gene‐importance scores revealed that SYNPO2 was the most important gene (Figure S3). SYNPO2 is a gene that encodes for the syntrophin‐2 protein, which plays an important role on the cell membrane. Recent studies have shown that the expression of SYNPO2 is closely related to the proliferation, migration, and invasion of cancer cells in a variety of tumours.^20^, ^21^ Through immunohistochemistry, we found that SYNPO2 is mainly expressed in the tumour cells, fibroblasts, inflammatory cell (eosinophils and lymphocytes) in gastric cancer tissue. However, SYNPO2 is expressed at nearly undetectable levels in the stromal cells of normal gastric mucosa (Figure 7). Additionally, SYNPO2 expression in gastric cancer tissue with peritoneal metastasis is significantly higher than that without peritoneal metastasis. These results suggest that SYNPO2‐positive expression in tumour cells, fibroblasts, inflammatory cell may be associated with promoting peritoneal metastasis in gastric cancer. These results imply that SYNPO2 may have a crucial function in the onset of GCPM and could be a useful tool for identifying this condition.

## 4 DISCUSSION

As a prevalent gastrointestinal tumour, gastric cancer poses a significant health threat to individuals worldwide.^22^ Unfortunately, patients diagnosed with advanced gastric cancer are particularly vulnerable to peritoneal metastasis following surgical removal, which is often indicative of a poor long‐term prognosis.^23^, ^24^ Metastases to the peritoneum are a major contributor to the high mortality rate associated with gastric cancer. Unfortunately, current treatment options tend to have limited efficacy in managing this condition, including chemotherapy, intraperitoneal infusion, and palliative surgery. In addition, early detection of peritoneal metastases in gastric cancer patients can be challenging when ascites are present in small amounts, or the metastases are hidden from view. Traditional methods of detection, such as imaging examinations or biopsies, may prove ineffective at improving the detection rate in these cases. This emphasizes the critical requirement for discovering novel predictive indicators that can detect peritoneal metastasis at an early stage, which would significantly enhance the likelihood of successful diagnosis and treatment of peritoneal metastases in individuals with gastric cancer.

This study involved the analysis of gene expression differences between GCPM and non‐GCPM, leading to the identification of a gene set consisting of five genes. We subsequently conducted an analysis of these genes, examining their correlations and other relevant characteristics. The analysis demonstrated that the five genes were linked to the EMT process. As EMT is known to be a key factor in the metastatic potential of tumour cells, this finding suggests that these genes may play a role in promoting metastasis.

Numerous studies have highlighted the critical link between gene mutations and tumour metastasis. Additionally, elevated expression of tumour neoantigens has emphasized the significant role of TMB in enhancing the response of cancer immunotherapy.^25^ It has been reported that TMB is inversely correlated with tumour metastasis due to the switch of MLH1 from silencing to activation.^25^ Gastric cancer's MSI molecular subtype is distinguished by a greater frequency of mutations and hypermethylation of the MLH1 promoter.^26^ When tumour metastasis takes place, MLH1 is activated to preserve the integrity of the DNA repair system, leading to decreased mutation rates. The findings of our study indicate that subtypes with low GCPMs had elevated somatic mutation rates and TMBs, whereas the subtypes with high GCPMs demonstrated the opposite pattern. Furthermore, several studies have consistently demonstrated that individuals with EBV positive subtype gastric cancer are more responsive to PD1 inhibitors like pembrolizumab.^27^ Our investigation revealed that the EBV positive subtype exhibited notably lower GCPMs in contrast to the EBV negative subtype. These findings provide additional evidence supporting the use of GCPMs as a robust framework for classifying patients with gastric cancer.

TME can significantly affect not only the advancement of tumours but also the response to treatment and overall clinical outcomes. CAFs located within the tumour stroma play a crucial role in facilitating the development of cancer cells. They achieve this by promoting extracellular matrix (ECM) deposition and remodelling, engaging in extensive communication with cancer cells, inducing EMT, enhancing invasion and metastasis, and conferring resistance to therapy.^28^ CAFs have been found to secrete cytokines such as HGF and OPN in colorectal cancer, which induce EMT. Additionally, CAFs release Wnt10b in exosomes, activating the Wnt/β‐catenin pathway and promoting EMT, ultimately facilitating metastasis of breast cancer cells. In the cases of lung and gastric cancers, CAFs release IL‐6, which has been found to enhance the migration and invasion of cancer cells while also inducing the expression of genes linked to EMT and metastasis. Research has indicated that CNN1 can heighten the level of matrix stiffness facilitated by CAFs, leading to chemoresistance in gastric cancer.^29^ Increased expression of OGN has been observed in ovarian carcinoma and has been found to exhibit a correlation with EMT signature and poor prognosis.^30^ By thoroughly investigating the changes in TME characteristics that arise from different GCPMs patterns, our study has demonstrated a clear association between GCPMs signatures and alterations in TME, specifically with regards to immune infiltration.

Subsequently, our study delved into the potential of GCPMs as a prognostic factor in the management of gastric cancer. Of note, immune checkpoint inhibitors are currently one of the most extensively researched forms of immunotherapy. Studies have shown that strategies involving checkpoint inhibitors targeting PD1, PDL1 and CTLA4 have exhibited superior overall survival outcomes when compared to traditional chemoradiotherapy approaches.^31^ There is a general consensus that EMT has an adverse impact on the response to immunotherapy and is a key contributor to poor survival rates in gastric cancer. This is mainly due to its ability to impair drug delivery to the tumour core by altering the TME, as well as reducing the tumour's susceptibility to treatment through immune evasion mechanisms.^32^, ^33^, ^34^ Our research findings indicate that a higher frequency of mutations in low GCPMs led to an upregulation of immune checkpoint genes. Additionally, we observed a positive correlation between GCPMs and CAFs, and patients classified under the GCPMs‐low subtype tended to show better responses to immunotherapy. The aforementioned findings collectively suggest that GCPM is a valuable predictive tool for precise immunotherapy in the context of gastric cancer.

Among the five genes screened as GCPMs biomarkers, the random forest model identified SYNPO2 as the most important gene for diagnosing GCPM patients. The results of immunohistochemistry suggest that SYNPO2‐positive expression in tumour cells, fibroblasts, inflammatory cell may be associated with promoting peritoneal metastasis in gastric cancer.

Despite potential clinical implications of our study, there are several limitations that need to be acknowledged. First, although we identified a set of DEGs and developed the developed the GCPMs, the underlying biological mechanisms responsible for the association between these genes and GCPM remain unclear. Further functional experiments and mechanistic studies are warranted to elucidate the specific roles of these genes in the development and progression of GCPM. Second, it is important to note that the identification and validation of GCPMs were based on retrospective data analysis. Prospective studies with larger patient cohorts and longer follow‐up periods are needed to confirm our findings and evaluate the clinical utility of GCPMs in guiding treatment decisions for gastric cancer patients.

## 5 CONCLUSION

In conclusion, we created a gene set score for GCPMs to classify patients and investigated the underlying pathways responsible for the disparate features observed in high versus low GCPMs samples. Our findings suggest that GCPMs has the potential to stratify patients, enabling identification of those who would derive greater benefit from adjuvant therapy and immunotherapy. Furthermore, it may facilitate the discovery of novel strategies and targets for cancer treatment. By incorporating the latest findings from genomic biology in GCPM, we can develop new and more effective peritoneal‐specific therapeutic strategies to improve outcomes for patients with this unique clinical entity.

## AUTHOR CONTRIBUTIONS

**Yuqin Sun:** Conceptualization (equal); data curation (equal); writing – original draft (equal). **YueQing Chen:** Methodology (equal); resources (equal). **Wei Zhuang:** Data curation (equal); formal analysis (equal). **ShunYong Fang:** Data curation (equal); investigation (equal). **QiuXian Chen:** Formal analysis (equal); methodology (equal). **MingQiao Lian:** Investigation (equal); methodology (equal). **ChenBin Lv:** Data curation (equal); formal analysis (equal). **JianMing Weng:** Methodology (equal); software (equal). **Ran Wei:** Software (equal); writing – original draft (equal). **Yao Lin:** Writing – original draft (equal); writing – review and editing (equal). **Lisheng Cai:** Conceptualization (equal); writing – original draft (equal); writing – review and editing (equal). **Qingshui wang:** Conceptualization (equal); data curation (equal); writing – original draft (equal); writing – review and editing (equal).

## FUNDING INFORMATION

This work was supported by the fund of Natural Science Foundation of Fujian Province (2020J01311402 and 2020J011301), the National Science Foundation for Young Scientists of China (82003095), the Startup Fund for Scientific Research, the Fujian Medical University (2018QH1203), The climbing Fund of PhD workstation, Zhangzhou Affiliated Hospital of Fujian Medical University(DPA202101), and the Science and Technology Development Fund of Macau SAR (FDCT) [0010/2021/AFJ and 0027/2022/A1].

## CONFLICT OF INTEREST STATEMENT

The authors have disclosed that they have no significant relationships with, or financial interest in, any commercial companies pertaining to this article.

## Supporting information


Figure S1.

Figure S2.

Figure S3.

## Data Availability

The datasets used to support the conclusion of this study were collected from publicly available databases including TCGA (https://portal.gdc.cancer.gov/) and GEO database (https://www.ncbi.nlm.nih.gov/geo/).
