# Supplementary figures and images for: Gastric cancer peritoneal metastasis related signature predicts prognosis and sensitivity to immunotherapy in gastric cancer
Source: J Cell Mol Med. 2023 Aug 21;27(22):3578–90. doi: 10.1111/jcmm.17922 (PMC10660625; doi:10.1111/jcmm.17922)

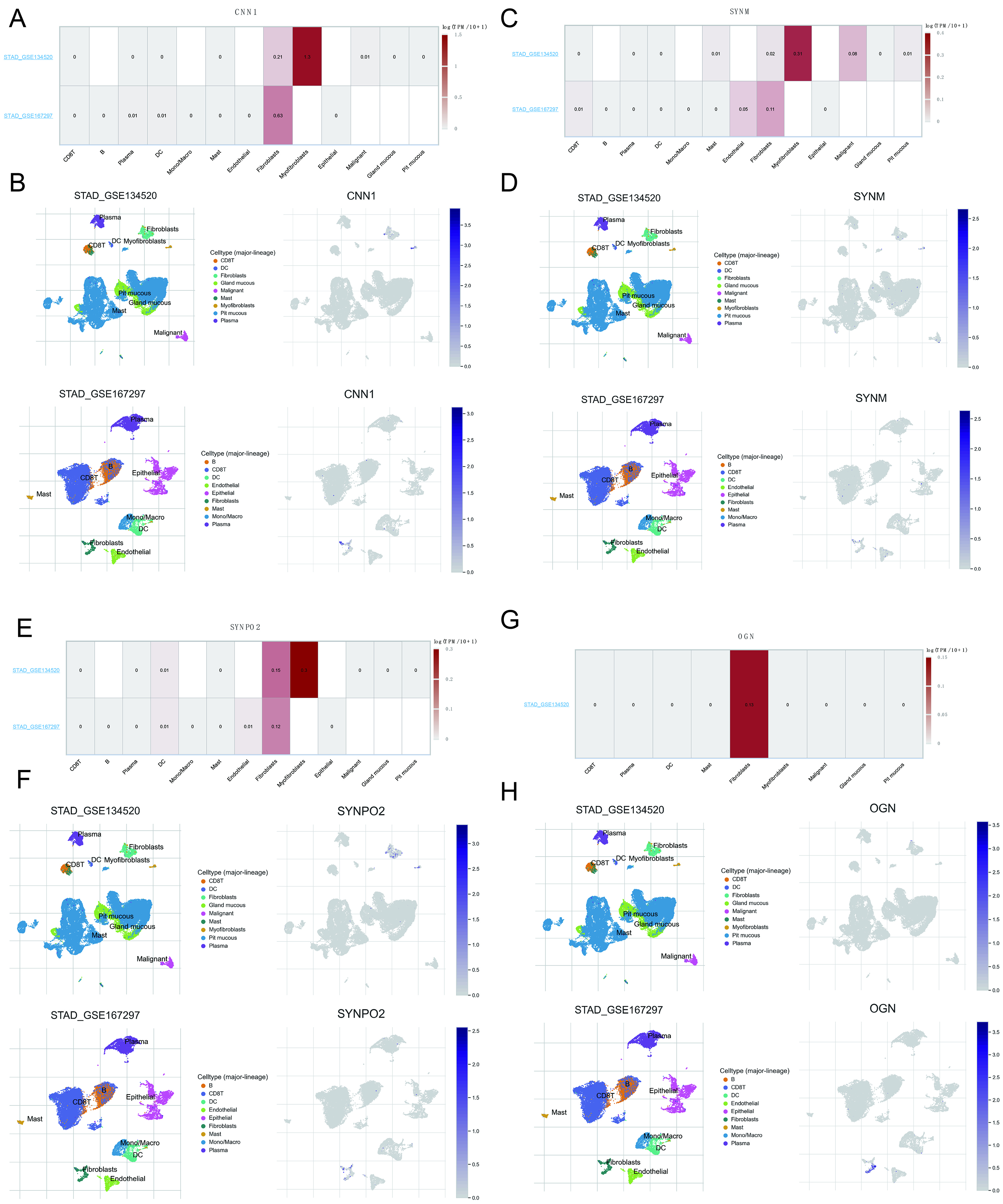

Supplement: Supplementary file 1 — Figure S1. [file JCMM-27-3578-s002.jpg]

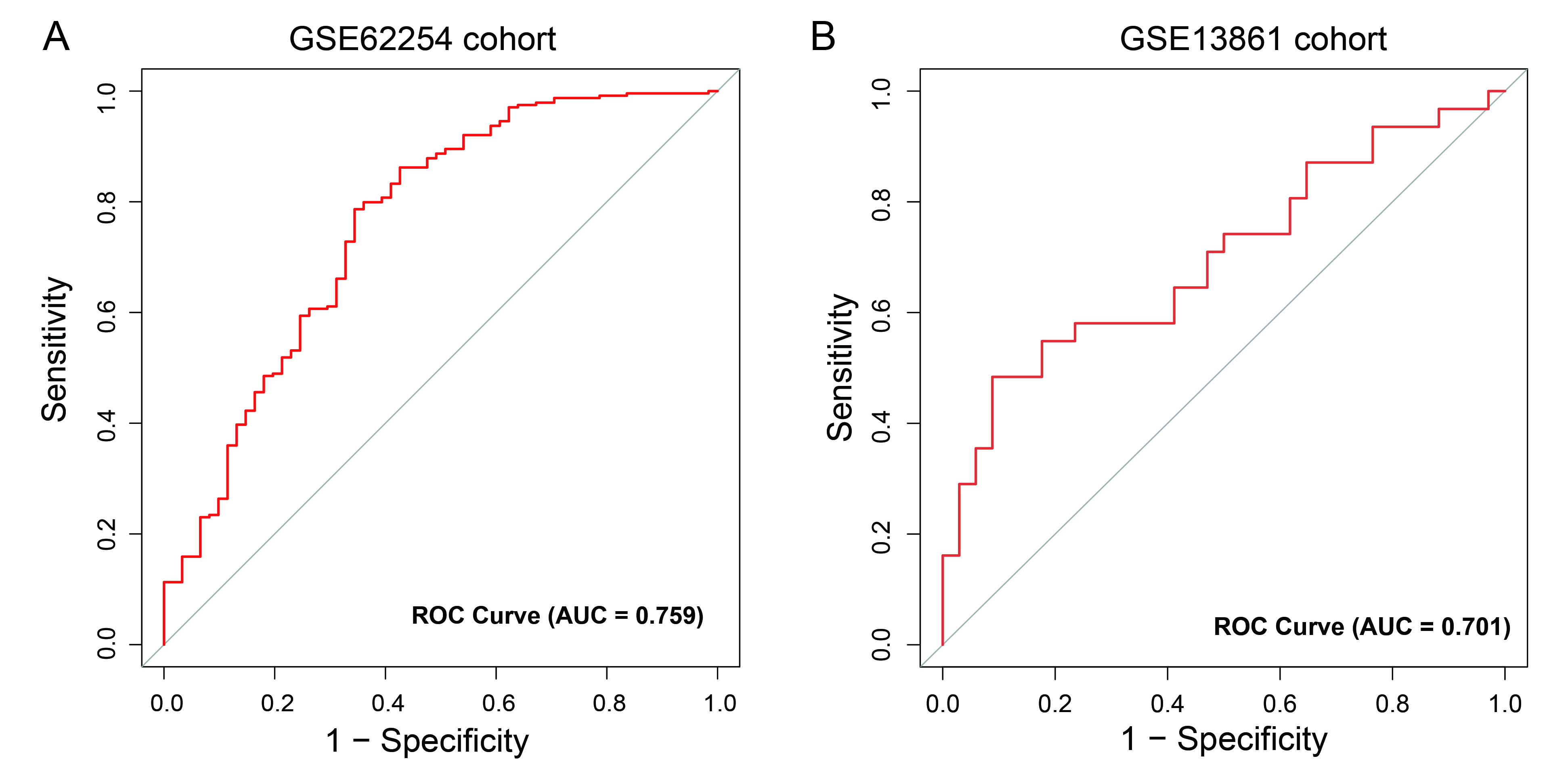

Supplement: Supplementary file 2 — Figure S2. [file JCMM-27-3578-s001.jpg]

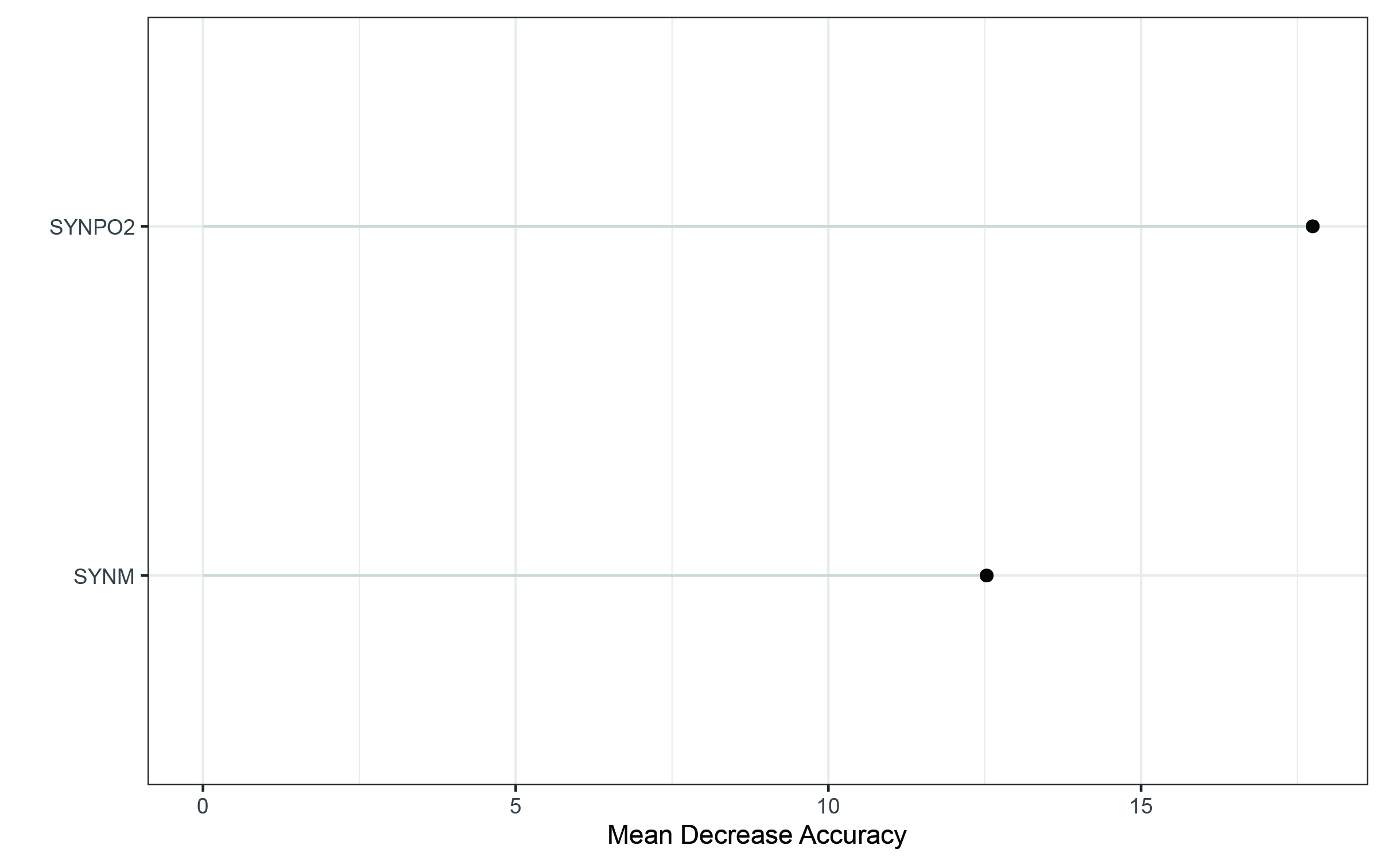

Supplement: Supplementary file 3 — Figure S3. [file JCMM-27-3578-s003.jpg]
